# Wearable Textile Platform for Assessing Stroke Patient Treatment in Daily Life Conditions

**DOI:** 10.3389/fbioe.2016.00028

**Published:** 2016-03-23

**Authors:** Federico Lorussi, Nicola Carbonaro, Danilo De Rossi, Rita Paradiso, Peter Veltink, Alessandro Tognetti

**Affiliations:** ^1^Research Center E. Piaggio, University of Pisa, Pisa, Italy; ^2^Information Engineering Department, University of Pisa, Pisa, Italy; ^3^Smartex, Pisa, Italy; ^4^Biomedical Signals and Systems, MIRA – Institute for Biomedical Technology and Technical Medicine, University of Twente, Enschede, Netherlands

**Keywords:** stroke rehabilitation, wearable sensors, data fusion, reaching, grasping, gait, ambulatory monitoring

## Abstract

Monitoring physical activities during post-stroke rehabilitation in daily life may help physicians to optimize and tailor the training program for patients. The European research project INTERACTION (FP7-ICT-2011-7-287351) evaluated motor capabilities in stroke patients during the recovery treatment period. We developed wearable sensing platform based on the sensor fusion among inertial, knitted piezoresistive sensors and textile EMG electrodes. The device was conceived in modular form and consists of a separate shirt, trousers, glove, and shoe. Thanks to the novel fusion approach it has been possible to develop a model for the shoulder taking into account the scapulo-thoracic joint of the scapular girdle, considerably improving the estimation of the hand position in reaching activities. In order to minimize the sensor set used to monitor gait, a single inertial sensor fused with a textile goniometer proved to reconstruct the orientation of all the body segments of the leg. Finally, the sensing glove, endowed with three textile goniometers and three force sensors showed good capabilities in the reconstruction of grasping activities and evaluating the interaction of the hand with the environment, according to the project specifications. This paper reports on the design and the technical evaluation of the performance of the sensing platform, tested on healthy subjects.

## 1 Introduction

Stroke survivors are trained to recover adequate control over their movements in order to optimize functional performances. Continuous daily-life monitoring of the functional activities of stroke patients in their physical interaction with the environment is essential for optimal guidance of rehabilitation therapy by medical professionals and for patient coaching. Such performance information cannot be easily obtained in usual conditions with current commercial monitoring systems. We introduce the INTERACTION sensing platform that was specifically designed and developed for ambulatory monitoring of post stroke patients. The INTERACTION modular prototype implements several functionalities aimed at a thorough study of the patients’ movements during daily activities. It combines a system for the movement estimation of the main joints of the human body, based on inertial sensing units (IMU) and knitted piezoresistive fabric sensors (KPF) in order to evaluate the recovery, as outlined in Veltink and Rossi (2010). The system is endowed with force sensitive resistors (FSR) placed on the hand and under the foot to estimate the force interaction with the environment. The movement tracking system has a set of textile based electrodes for the electromyography (EMG) acquired on the deltoid muscle. The integration of this system of sensors, fused by dedicated algorithms, completes the set of information for a general overview of the physiological/pathological quality of movement during normal activities at home, after a clinical rehabilitation period (Veltink et al., 2014). The minimal sensor set, which is less cumbersome, led to an unobtrusive measurement system thanks to the large number of textile sensors used. This system can be worn without discomfort for long monitoring periods. Inertial measurement units, based on the integration of accelerometers, gyroscopes, and magnetometers, are commonly considered as the gold standard in motion tracking (Roetenberg et al., 2005; Liu et al., 2009; Sabatini, 2011). IMUs estimate the orientation of the body segments where they are attached by combining multi-sensor information through dedicated optimal sensor fusion algorithms, mainly based on Kalman filtering (Roetenberg et al., 2005; Sabatini, 2011). The general approach is to apply strap-down integration of the gyroscope signal (Savage, 1998) and to correct the inclination and heading drifts through accelerometer and magnetometer measurements. The combination of different IMUs, placed on connected body segments, and the additional information on the kinematic constraints enable selected joint angles to be measured (Luinge et al., 2007; Roetenberg et al., 2007). Although they are commonly used in this field, IMUs suffer from a loss of accuracy due to magnetic disturbance, they can be bulky and expensive. The number of inertial units included in the INTERACTION devices is, thus, minimal and they have been replaced, where possible, by textile sensors. We fused information derived from IMUs with data from KPF sensors, EMG electrodes, and force sensors. Knitted piezoresistive fabric, which is widely presented and characterized in Pacelli et al. (2006, 2013), Carbonaro et al. (2014), and Dalle Mura et al. (2014) Tognetti et al. (2014b), has promising properties, such as short transient time, reduced aging, washability, and reproducibility. It has been used both as strain sensors and, arranged in double layer devices, such as goniometers to monitor joint movement. Thanks to its unobtrusiveness, the textile integration can be improved. To implement a robust home-monitoring system and reduce the encumbrance, the acquisition electronics aimed at acquiring data from KPF were integrated into the IMU chassis, and designed *ad hoc*. The textile electrodes were implemented using woven conductive fibers, elastic fibers, and natural or synthetic yarns, as reported by Caldani et al. (2013). Section 2 describes the sensing platform. The set of IMUs and textile sensors were conceived as a compromise between patient comfort and the fulfillment of the clinical requirements defined by stroke professionals (i.e., the least obtrusive sensing system capable of evaluating the patient residual performance of upper and lower limbs). Section 3 describes the materials employed, the sensors used, and their working principles, focusing on textile wearable sensing and electromyography electrodes, complete with a characterization of their mechanical and electrical properties. Section 4 describes the modules that make up the INTERACTION prototype with particular focus on the integration methods of the textile and conventional sensors. The aim of this paper is to demonstrate the technical feasibility of the INTERACTION full body monitoring prototype in terms of evaluating the residual functionalities of the stroke patient. Thus, the validation trials were performed on healthy people to prove the adequate performance of the modular prototype (sensors, related fusion, and movement reconstruction methods). Special attention was focused on the upper limb (reaching, section 4.1), hand (grasping, section 4.3), and lower limb (gait, section 4.2). Clinical tests on stroke patients are out of the scope of this work and were performed – using the presented technological platform – in a subsequent phase of the research as presented by van Meulen et al. (2016).

## 2 The Interaction Wearable Sensing Platform

The INTERACTION project aims to implement a sensing platform to acquire information on the residual movement functionality in patients after a stroke. In order to improve the patient’s mobility, all the residual body activities are important and must be taken into account. The sensing system, thus, covers the whole body, except for the head. The aspects we considered in the design of the platform include the functionality of the sensors, sensing capability, and connectivity to an acquisition portable device. In parallel, we ran a design study by considering the users requirements in terms of comfort and usability. Patients that recover from a stroke and more generally, subjects that present movement impairments, cannot easily wear, take off a garment or adjust the position of the sensors on their body. Hence, the design was based on the concept that the system components need to be put on and taken off using only the unaffected part of the body, with the help of a residual functionality of the affected part. The system was designed and developed by a team of engineers, clinicians, and tailors to satisfy the technical specifications (i.e., sensor placement, functionalities, accuracy, aging), comfort, and use requirements, paying particular attention to the condition of people who have suffered a stroke. Under these guidelines, and according to the patient’s needs, the sensor system was developed in four different modules that can be worn separately: a sensing shirt, a pair of sensing trousers, a kinesthetic-kinetic glove, and a sensing shoe. Three different types of textile sensors were integrated in the system: textile electrodes for the EMG measurements, piezoresistive fabrics, and textile goniometers. All these sensors need to guarantee that their position on the patients body will not change during their use. The textile sensing system was supplemented by a minimal set of inertial measurement units (IMUs) to track the orientation of body segments and force sensitive resistors (FSRs) in order to evaluate the interaction with the environment.

The functionalities required for the sensing system mainly consisted of:

- monitoring of reaching distance and quantifying frequency and type of grasp of the affected and unaffected arms;
- detecting sEMG for quantifying shoulder abductor effort and detecting possible pathological/compensatory synergies; and
- estimating weight distribution and kinematic asymmetries between affected and unaffected legs in gait/ambulation.

**Figure 1:**
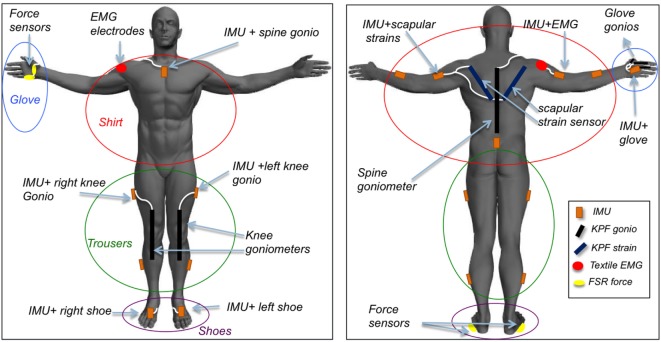
**Textile-integrated sensing system for daily-life assessment of motor performance in stroke patients, including inertial sensor modules on main body segments, shoulder abductor EMG, shoulder strain sensing, spine and hand goniometers, shoe, and glove force sensing**. The system is divided into shirt, trousers, shoes, and gloves. Patient-specific combinations of modules can be chosen.

**Figure 2:**
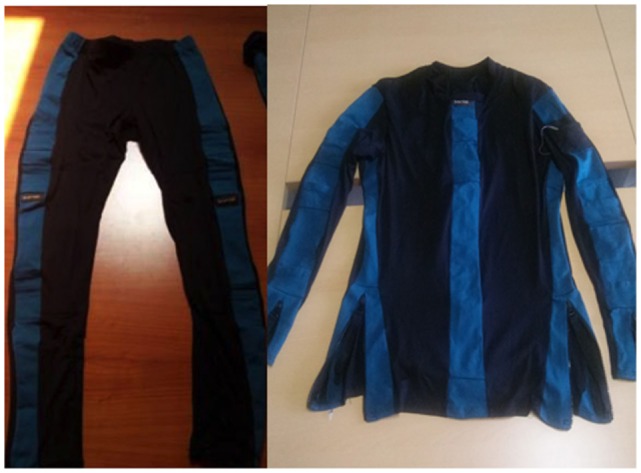
**The trousers and shirt modules of the INTERACTION prototype**.

The specifications of the sensing system are illustrated in Figure 1. The shirt was endowed with five IMUs, placed on the arms, forearms, and sternum (orange boxes). These sensors reveal the flexion of the trunk and the orientation of the humerus and the radius during patient activities. To take into account the movement of the scapular-thoracic joint, the set of kinematic sensors used for the scapular girdle monitoring was supplemented by a couple of strain sensors (blue stripes in the figure) placed between the spine and the acromion area, aimed at revealing and measuring scapular sliding during the arm movement. To integrate the information on the trunk flexion, a textile goniometer (black stripe in Figure 1) was placed along the spine. Finally, an EMG electrode was placed on the deltoid to estimate the muscle contraction in anti-gravitational actions (red circle in Figure 1). The orange boxes containing the IMUs, fixed in *ad hoc* pockets on the garments, are endowed with radio devices that enable them to be connected with a central unit gathering pre-processed signals for further elaboration. Each peripheral radio device has a channel for sending data derived from external (i.e., KPF, EMG, force) sensors. Thus, each textile sensor is wired to a IMU (white path, in the figure). Similarly, the trousers were equipped with a couple of IMUs per leg, placed on femoral thigh and, laterally on the calf. A textile goniometer, placed on the knee to increase the information derived from the inertial system was connected to the IMU box on the hip. The sensing set of the trousers also has an IMU placed on the sacrum that is used, along with the spine goniometer and the sternum IMU, to reconstruct the global orientation of the trunk with respect to the pelvic girdle. The trouser sensors are shown in Figure 1 with the same symbols used for the shirt. The INTERACTION shirt and trousers are also shown in Figure 2. The glove module was developed for the ambulatory evaluation of the residual hand function and its recovery. The main requirements of the hand sensing system – identified through questionnaires and interviews held with professionals and patients (Tognetti et al., 2014a) – were the quantification of the frequency and type of grasp of the affected and unaffected arms during daily activities. The objective was to perform a continuous classification and discrimination from the set of basic functional hand grips defined by Lister (1977) and referring to common interaction tasks with the external environment. An additional requirement was the evaluation of the stroke patient force interaction with the environment aimed at quantifying the use of upper extremities to support body weight during daily activities (e.g., hand support in standing up tasks). The glove is equipped with three goniometers placed on the thumb, forefinger, and middle finger, which cooperate with a force sensor (yellow circle in Figure 1) placed on the lateral part of the forefinger to distinguish the hand poses, and an IMU aimed at reconstructing the hand orientation with respect to the sternum in monitoring reaching and grasp activities. In the INTERACTION project, we developed both left and right hand gloves to simultaneously monitor the affected and unaffected arms. In addition, the distal part of the fingers was left uncovered in order to preserve touch sensation. Finally, the instrumented shoe, whose core is constituted by a force-sensing sole, was provided by an IMU to detect the foot orientation in order to evaluate the interaction with the environment of the individual foot and the balance of the whole body during standing position and gait.

## 3 Materials

In this section, the commercial and custom-developed sensors used to develop the INTERACTION prototype are described and their working principles discussed.

### 3.1 Wearable Inertial Sensing

The inertial measurement units provided by Xsens (MTw) are currently used in their MVN suite^1^. The Xsens MVN motion capture suit is an easy-to-use system for full body motion capture. MVN is based on miniature inertial sensors, biomechanical models and sensor fusion algorithms. The MVN model consists of 23 segments with 22 joints. Each joint is specified by statistical parameters for 6DOF joint laxity. Movements are captured by 17 motion trackers. Each motion tracker has a 3D orientation accuracy of less than 2° RMS (when the MVN suit is used in a homogeneous magnetic field). The resolution of each motion tracker is 0.05°. The sensor range of the 3D accelerometer is 180 m/s^2^ (18 g), and the sensor range of the 3D gyroscope is 1200 deg/s. The system has a flexible calibration scheme with instant feedback regarding the expected accuracy. The data are recorded by two Xbus Masters, which collect the data and send it to a remote PC through a Bluetooth connection. The maximum update rate is 120 Hz, and the typical battery operating time is 3 h. Although MVN is a powerful measurement system, it is not suitable to be worn by stroke patients for monitoring in their home situation and over a long time. The MVN suit is not unobtrusive, nor can it be put on by the patient without the assistance of other people. Thus, the strip bands that support the IMUs were replaced by pockets sown onto the sensing garments. The electronics that acquire the data from inertial sensors were integrated with acquisition systems aimed at capturing information from textile and force sensors. The same wireless connection was also used to deliver these data to the remote PC that monitors all the system.

### 3.2 Wearable KPF Sensors

**Figure 3:**
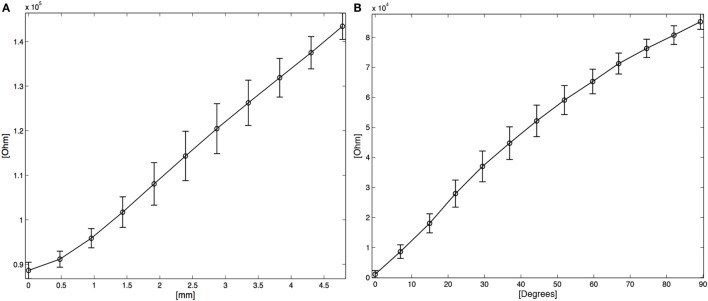
**(A)** Average R vs. applied deformation in length. **(B)** Average ΔR* vs. angle. The vertical bars represent two SD units in length.

**Figure 4:**
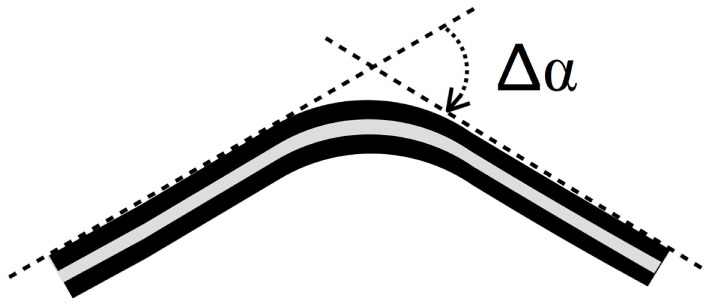
**A double layer KPF goniometer**. The black stripes represent the two identical piezoresistive layers, while the gray stripe is the insulating layer. When the sensor is in a flat position, the resistance difference (Δ*R*) between the two layers is 0. When the sensor is flexed Δ*R* is proportional to the bending angle (Δ*α*), defined as the angle between the tangent planes to the sensor extremities (green dashed line in the picture).

Textile-based sensors were created using knitted piezoresistive fabrics (KPF), which contain 75% electro-conductive yarn (Belltron^®^, produced by Kanebo Ltd.) and 25% Lycra^®^, manufactured as a single jersey in a circular knitting machine, as described in previous works (Pacelli et al., 2006, 2013). A conductive bicomponent fiber yarn based on polyamide loaded with carbon particles is used in combination with lycra. Piezoresistive fabric sensors change the electrical resistance according to the strain applied. The variation in the electrical properties is due to the change in the interconnection geometry inside the fabric structure. Usually this property can be observed in stretchable fabric where the elongation of the fibers affects the flow of carrier inside the structure. When the conductivity of the yarn is due to the presence of conductive particles as in bicomponent fibers, the elongation of the yarn affects the charge transport mechanisms. The interconnections among fibers and stitches are altered by the deformation. The elongation of the fabric modifies the distance between stitches as well as the arrangement of the fibers in the yarn leading to different interconnection geometry. Tognetti et al. (2014b) proves that the electrical resistance shown by a textile specimen is given by:

(1) $$R_{\mathrm{SL}}=l\frac{\rho }{d\;h}-\frac{\rho }{d}\;\Delta \alpha +O\left(\underset{s\in \left(0,l\right)}{\text{sup}}k{\left(s\right)}^{2}\right)=l^{2}\frac{\rho }{V_{0}}-\frac{\rho }{d}\;\Delta \alpha +O\left(\underset{s\in \left(0,l\right)}{\text{sup}}k{\left(s\right)}^{2}\right)$$

where *V*_0_ is the volume of the specimen, *l* is the actual length of the sensor, *d* is its width, Δα is the angle between the tangent planes to the sensor extremities, *h*_0_ is the initial thickness of the specimen, and *O*(*sup*(*k*(*s*)^2^)) is a second-order infinitesimal function that tends to 0 for *k*(*s*) → 0. Note that the curvature *k*(*s*) assumes high values only when the sensor has a rapid change in direction (as in cusps), which makes the function *O*(sup*_s_*_∈(0,_*_l_*_)_*k*(*s*)^2^) negligible in detecting the human body shape. In this work, the bending angle is in practice negligible, so only the piezoresistive component of equation (1) is considered to evaluate scapular sliding. The elongation of the KPF sensors was characterized using a custom-designed electro-dynamic testing system based on a linear motor controlled by a PLC and able to apply strain cycles with controlled amplitude and velocity. The numerical results hereby reported were obtained from tests performed on a specimen 20 cm in length, and 2 cm in width. Sensors with different dimensions have, however, shown the same behavior. A single-layer piezoresistive device was tested in order to estimate the linearity of the electromechanical characteristics and evaluate the inaccuracy introduced by hysteretic phenomena (Tognetti et al., 2014b). The average values and the SDs of the electrical resistance were calculated (as in equation (2)) on repeated elongation cycles for different lengths (as shown in Figure 3A) in order to create an electromechanical characteristic of the strain sensor:

(2) R¯i=1P∑pRi,pσRi=∑p 1PRi,p−R¯i2,

where *p* denotes the *p^th^* trial executed for a certain imposed length *i* and *P* the total number of trials. To roughly estimate the sensor electromechanical properties, ${\bar{R}}_{i}$ vs. deformation characteristic was approximated by a linear function and the deformation sensitivity (*S*) was computed as the slope of its linear approximation. The deformation sensitivity *S* is $11950\frac{\Omega }{\mathrm{mm}}$ and the maximum SD $\sigma_{\mathrm{Max}}=\max_{d}\sigma_{R_{d}}$ holds 5603Ω. As described in our previous studies (Carbonaro et al., 2014; Dalle Mura et al., 2014; Tognetti et al., 2014b) and reported in Figure 4, textile goniometers were developed by coupling two piezoresistive layers through an electrically insulating layer. The sensing layers were made of knitted piezoresistive fabrics. Ideally, if the two KPF layers were geometrically and electrically equivalent, the sensor output, represented by the resistance difference between the two sensing layers (Δ*R*) would vanish when in a flat position and would be proportional to the flexion angle (*θ*), except for a second-order infinitesimal function, as the local curvature tends to 0 (by subtracting two instances of equation (1)), one for each layer as in Lorussi et al. (2009):

(3) ΔR≃k Δα.

In practice, equation (3) is not verified, due to the real differences in the electrical properties between the two piezoresistive layers. In Tognetti et al. (2014b), it has been proved that the *θ* vs. Δ*R* relation can be reasonably approximated by the following linear function

(4) ΔR=sΔα Δα+ΔRo,

where *s_Δα_* and Δ*R*_0_ represent the goniometer sensitivity and offset, respectively. The angle values can be computed by equation (4) as:

(5) Δα=ΔR−ΔRosθ=c1 ΔR+c2.

In equation (5), parameters *c*_1_ and *c*_2_ had to be identified by experimental trials. It is, thus, necessary to perform a calibration procedure to determine them. According to equation (5), by acquiring Δ*R*_1_ and Δ*R*_2_ in two different angular positions Δ*α*_1_ and Δ*α*_2_, it is possible to compute *c*_1_, *c*_2_ as

(6) c1=Δα1−Δα2ΔR1−ΔR2c2=ΔR1 Δα2−ΔR2 Δα1ΔR1−ΔR2.

In the characterization reported in the following, before integrating the goniometer into the textile, a bench calibration in the angular positions (Δα_1_ = 0° and Δα_2_ = 90°) was preformed to obtain the *c*_1_ and the *c*_2_ values. Also in this case of bending, the mean (7) and the SD (8) of ΔR* were calculated and plotted as a function of the angle Δ*α* (Figure 3B).

(7) $${\bar{\Delta R^{*}}}_{\Delta \alpha_{i}}=\frac{1}{K}\sum_{k}\Delta R_{\Delta \alpha_{i},k}^{*}$$

(8) $$\sigma_{\Delta R_{\Delta \alpha_{i}}^{*}}=\sqrt{\sum_{k}\frac{1}{K}{\left(\Delta R_{\Delta \alpha_{i},k}^{*}-{\bar{\Delta R^{*}}}_{\Delta \alpha_{i}}\right)}^{2}}$$

${\bar{\Delta R^{*}}}_{\Delta \alpha_{i}}$ trend was approximated by a linear regression in the least-squares sense and the angular sensitivity of the double layer sensor *S*_Δα_*_DL_*, is estimated as 955 ^Ω^/°. The maximum SD was evaluated:

(9) σMax=maxΔαi σΔRΔαi∗=5100Ω

for Δα = 37°, corresponding to an angular error of 5.3°.

### 3.3 Wearable Textile Electrodes

**Figure 5:**
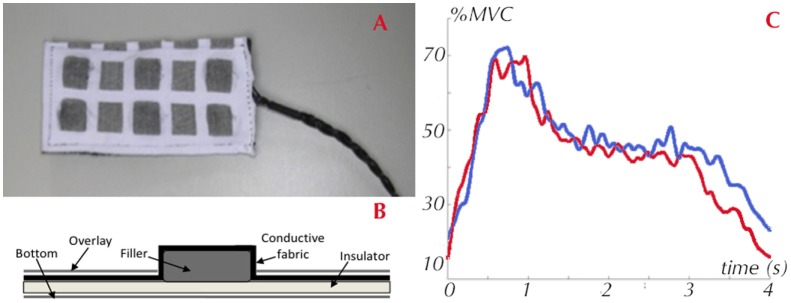
**Textile electrodes for electromyography signal acquisition: (A) Electrode matrix; (B) Electrode conceptual design; (C) sEMG signal gathered by the textile electrodes on the deltoid compared with the commercial system (Biometrics)**.

The investigation into the textile electrodes for surface electromyography (sEMG) detection focused on selecting appropriate materials, the textile process, and designing the electrode matrix. The shape, dimension, distance, and size of the electrodes were selected for preliminary tests according to literature standards (Hermens et al., 1999). The best performances were obtained by using electrodes created with conductive yarn based on stainless steel fibers (30%). The textile electrodes developed and the dedicated acquisition electronics were tested on several upper and lower body muscular groups in order to obtain information on muscular activation during rehabilitation therapy or daily activities, although in the final INTERACTION prototypes they were used only to detect the activity of the deltoid muscle. The textile electrodes used in the prototype for the acquisition of the sEMG signal of the deltoid muscle have a square shape (10 mm side) and a inter-electrode distance of 20 mm (Figure 5A) and are based on a stainless steel yarn. The electrodes were integrated in a non-conductive fabric composed of antibacterial nylon and elastane. In order to insulate the back of the electrodes and to improve the contact with the skin, a multilayer structure was used, composed of a layer of polyurethane foam, a back layer of neoprene, and an external layer of fabric (Figure 5B). The connection wires are made with textile-compatible cables, with a stainless fiber core and a PVC coating. To connect the textile sEMG electrodes with the rest of the sensing system, an electronic circuit was designed, developed, and connected to the upper arm IMU board (Figure 1 on the right). This compact acquisition unit extracts the EMG envelopes calculated through high-pass filtering, rectification, and low-pass filtering of the raw EMG. The EMG envelopes are then sent to the available digital channel of the upper arm IMU board.

Six healthy subjects were involved in the preliminary laboratory testing of the EMG system aimed at analyzing the textile electrode performance. The testers, four men and two women, had an average age of 35 years. The muscles investigated were: deltoid, biceps, triceps, wrist extensors, and flexors. The protocol consisted of a total of seven trials per measurement, which were repeated for each subject, each muscle and external load. The sEMG signal was acquired with the textile electrodes and was compared with a commercial EMG system considered as the gold standard in this analysis (Biometrics System (DLK400) with their proprietary pre-amplified electrodes). The results for the deltoid muscle are reported below. The testing protocols consisted in acquiring:

- Baseline, measured once at the beginning of the experiment, for 30 s.
- Maximum voluntary contraction (MVC), measured three times for 10 s (2 s resting, 6 s in MVC, and finally 2 s resting).
- Dynamic trial, lasting 8 s. The subjects were asked to rest for 4 s, then to lift their arm and hold the position for 2 s, and then to move back to the resting position.

This sequence was repeated 10 times per trial and for three different loads; 0 kg, 2 kg, and 4 kg. The data processing was performed as follows:

- subtraction of the mean value from the signals,
- perform a second-order Butterworth high pass filter at 10 Hz,
- perform a second-order Butterworth low pass filter at 300 Hz,
- rectification of the signal (absolute value), and
- normalization of the signals with respect to the MVC (to compare the SEMG signal with the gold standard).

The latter step (i.e., the classic MVC normalization) is necessary to facilitate a comparison between the two raw signals due to the different amplifications of the two EMG systems. After the normalization, we calculated the EMG envelopes using a second-order Butterworth filter at 6 Hz. Finally, the signal-to-noise ratio (SNR) was computed for each EMG system by considering the root mean square (RMS) of the active part of each MVC trial and comparing it with the RMS of the complete baseline trial, according to the following equations:

(10) SNRdB=20 logRMSSignalRMSBaseline,withRMS=1N∑i=1Nxi2

where *N* is the number of samples per trial. Average values of SNR for the two EMG systems are reported in Table 1. Figure 5 compares the signals typically acquired on the deltoid during the arm abduction where a good accordance between the two acquired signals is shown. Table 1 highlights that the textile electrodes do not perform as well as the standard ones (lower signal-to-noise ratio). Despite the lower signal-to-noise ratio, the textile electrodes guarantee the discrimination between the activation/deactivation phase of the deltoid muscle, thus respecting the requirements of the interaction platform.

**Table 1:** **SNR acquired by textile electrodes and compared with the gold standard (Biometrics)**.

Subject	Textile	Biometrics
*S*_1_	28.71	39.33
*S*_2_	22.86	35.93
*S*_3_	29.58	47.07
*S*_4_	23.72	33.50
*S*_5_	29.49	37.54
*S*_6_	27.21	39.29

### 3.4 Force Sensors

The FlexiForce force sensor selected for the INTERACTION prototype is a commercial device produced by Tekscan. The sensor is an ultra-thin, flexible printed circuit. The standard A201 force sensor is constructed from two layers of substrate (polyester) film. The high-temp model (HT201) is constructed from two layers of polyimide. On each layer, a conductive material (silver) is applied, followed by a layer of pressure-sensitive ink. Adhesive is then used to laminate the two layers of substrate together to form the force sensor. The active sensing area is defined by the silver circle on top of the pressure-sensitive ink. Silver extends from the sensing area to the connectors at the other end of the sensor, which form the conductive leads. The FlexiForce sensor acts as a force sensing resistor in an electrical circuit. When the force sensor is unloaded, its resistance is high. When a force is applied to the sensor, this resistance decreases. In order to integrate the FlexiForce sensor, a force-to-voltage circuit was designed. A means of calibration must then be established to convert the output into an appropriate discrete force status. The acquisition system was designed to implement the specifics, which consists in discriminating three force levels as follows:

- zero force, for forces smaller than 1 N,
- low force, for forces between 1 and 10 N, and
- high force: for forces that exceed 10 N.

This was done to implement the kinetic specifications of the glove, reported in Section 4.3.

## 4 Methods and Results

In this section, the modules of the INTERACTION prototype are described by focusing on the integration of the information derived from the textile sensors with the information from the conventional sensors. For each prototype, the results of the experiment trials performed on healthy people for the functionality validation are reported.

### 4.1 Module 1: Sensing Shirt

Objective and patient-specific performance assessments of daily arm movements are fundamental for an optimal guidance in neurological rehabilitation therapy. An accurate estimation of the hand position with respect to the sternum is fundamental in evaluating the recovery induced by the treatment. In most applications, the shoulder movement is simplified through a socket-ball representation and neglecting the scapular-thoracic complex. In the sensing shirt developed within the INTERACTION project, we propose an innovative sensing configuration to estimate the hand vs. sternum position. This fuses the information of inertial sensing and textile strain sensors to take into account the complexity of the scapular-thoracic and the gleno-humeral movements.

#### 4.1.1 Sensing Shirt: Methods

In this paper, data derived from IMUs placed on the arm and the sternum of the affected side, a KPF strain sensor placed on the scapula, and an EMG electrode on the deltoid are considered to improve the reconstruction of the position of the hand with respect to the sternum. Since the aim of this section is to describe the improvement in accuracy of the reconstruction of the shoulder movement contributing to hand reaching, we consider the trochlea of the humerus as the end effector. In the case of a completely extended elbow, the position of the hand with respect to the gleno-humeral joint is simply obtained by re-scaling the position of the trochlea along the entire length of the arm. By thus considering a reduced length for the arm in movement acquisition enabled us to obtain biomechanical data in a more compact space, thus increasing the acquisition reliability of the optical system [Smart DX 100 produced by^2^] used as a gold standard to calibrate and validate the method. In order to improve the hand position estimation, the following steps were implemented:

- A set of experiments was set up with the optical system used both for identifying the parameters of a bi-articular model of the shoulder on particular trajectories of the arm and subsequently to validate the model as a gold standard measurement system as follows:
- biomechanical variables were obtained during arm abduction and arm flexion on the sagittal plane using the INTERACTION shirt,
- the trajectories of the trochlea were reconstructed using both the optical system proprietary software and a simple socket ball model fed by the IMUs alone,
- the two reconstructions were compared to analyze and mathematically remove the movements of the trunk,
- a bi-articular model for the shoulder, taking into account the scapular movement on the rib cage, was proposed in a parametric form,
- the parameters of the new model, valid for the entire shoulder workspace, were identified using the data acquired on the two movements considered, and
- The 3D position reconstruction of the trochlea performed by the bi-articular model was compared with the gold standard optical system to estimate the assessment of the end effector coordinate.

To create a reliable shoulder model capable of estimating the hand position, we asked five healthy subjects to perform plane movements of the shoulder and we collected the data derived from the sternum and arm IMUs and strain sensor placed on the back (from spine to scapula). Simultaneously, the 3D positions of repere points needed to reconstruct the movement of the scapular girdle (sternum, acromion, medial epicondyle of the trochlea of humerus) were acquired by the optical system. An initial data analysis was performed to develop the model so that could mimic the scapular girdle for the two planar movements considered:

- an abduction of the arm on a frontal plane in the range of 0–150° from the anatomical position, and
- a flexion on a sagittal plane of the arm in the range of 0–120° from the anatomical position.

**Figure 6:**
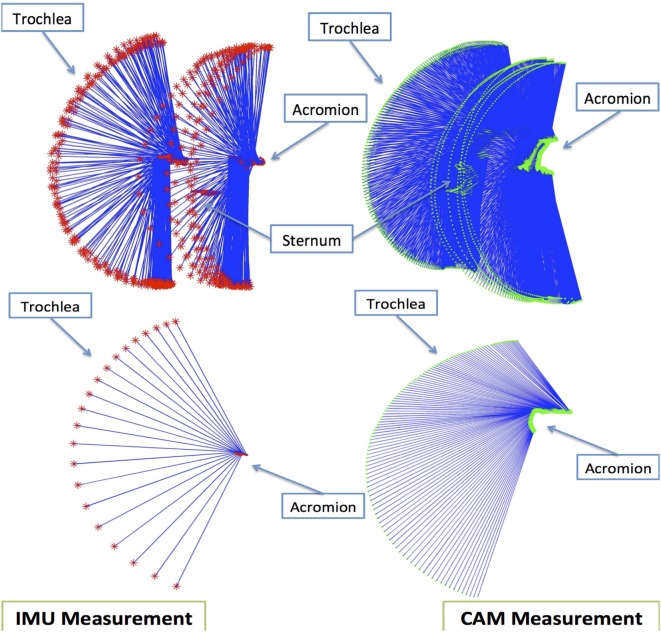
**Textile-integrated sensing system for daily-life assessment of motor performance in stroke, including inertial sensor modules on main body segments, shoulder abductor EMG, shoulder strain sensing, spine and hand goniometers, shoe, and glove force sensing**. The system is divided into shirt, trousers, shoes, and gloves. Patient-specific combinations of modules can be chosen.

**Figure 7:**
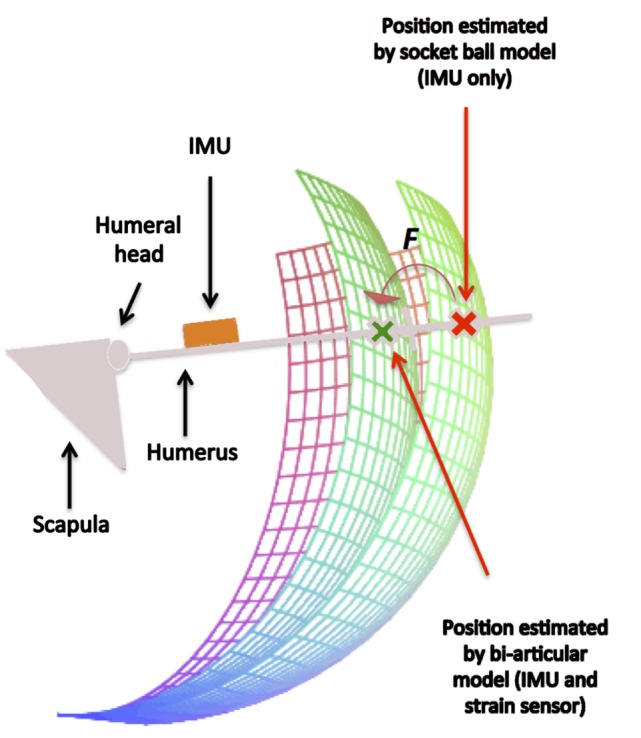
**The action of the map *F* derived from the bi-articular model for the shoulder**. The estimated position of the trochlea, initially lying on a sphere according to the socket ball model, is improved taking into account the global movement of the scapular girdle.

It is known from kinesiology (Kapanji, 1983) that the first movement is composed of the inference of two different movements, an abduction of the scapular-humeral joint of 90° and a rotation of the scapula on the rib cage of 60°, approximately. On the other hand, using a simple socket-ball model, the two movements are commonly interpreted as a circle arc. Similarly, the flexion on a sagittal plane results in the combination of a flexion of the gleno-humeral joint of about 60° combined with a depression of the scapula on the rib cage leading to a global orientation of 120° with respect to the craniocaudal axis. Again, using the socket-ball model, the movements are reconstructed as a circle arc of the trochlea. The two trajectories gathered by the IMUs are shown in Figure 6. The socket ball model reconstruction is represented on the left, while the model obtained by the optical system is represented on the right. In both cases, the upper pictures derived from the data refer to an inertial frame that is fixed with respect to the environment. In order to estimate the position of the trochlea with respect to the sternum reference, oscillations of the thorax due to the movement were removed in both cases. The resulting data were plotted in the lower part of Figure 6 where a monolateral case is considered. As expected, the socket ball model reconstructed a circle as a trajectory, and the humeral head position did not change with respect to the sternum. On the other hand, the real trajectory plotted on the right showed movements of the acromion and, consequently, of the humeral head. In our bi-articular model, the humeral head trajectory was approximated by an elliptic arc, both for flexion and abduction movements. The trochlea trajectories instantaneously created a circle arc whose center moves on the humeral head path. According to this approximation, the proposed 3D model for the shoulder movement was developed as described below. The status manifold of the positions of the trochlea is corrected starting from the spherical status surface of the socket-ball model into a different manifold obtained by considering a circular evolution (whose radius is equal to the humerus length) with a center capable of moving on an ellipsoid. Data derived from the strain sensor, placed on the scapula, were then used to operate the correction from the sphere to the bi-articular status manifold, as represented in Figure 7. To summarize, the function *F* shown in Figure 7 is an iterative map formally represented as:

(11) $$F=\left(\begin{array}{c} \theta_{\mathrm{arm}}\\ \psi_{\mathrm{arm}}\\ s \end{array}\right)\mapsto \left(\begin{array}{c} X_{\mathrm{end}.\mathrm{eff}}\\ Y_{\mathrm{end}.\mathrm{eff}}\\ Z_{\mathrm{end}.\mathrm{eff}} \end{array}\right),$$

where *θ_arm_* and *ψ_arm_* represent the flexion on an horizontal plane and abduction angles of the arm, respectively, detected by the IMU placed on the upper arm with respect to the IMU fixed on the sternum, while *s* indicates the value of the strain sensor.

#### 4.1.2 Sensing Shirt: Results

**Figure 8:**
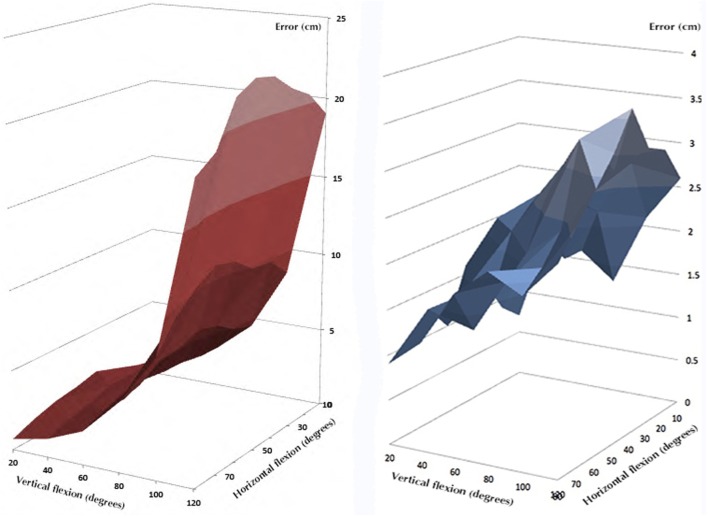
**The average errors committed by the two approaches with respect to the gold standard**. The blue surface represents the error introduced by the bi-articular model, while the red surface is related to the socket-ball and IMU estimation.

The performances of *F* in reconstructing the position of the hand were tested by a series of vertical movements on different planes. The results of the estimation were compared with the output of the optical system used as a gold standard reference system. Table 2 reports the mean absolute errors with respect to the position reconstructed by the optical system. The same data are represented in Figure 8. The maximal value of the average error is obtained for 40° horizontal flexion and 120° abduction and the difference with the gold standard instrument is 3.6 cm. The trend of the bi-dimensional error map is approximately symmetrical with respect to the path characterized by 40° horizontal flexion, which contains (see Table 2, forth column) the maximal values for fixed abduction angles. Figure 8, on the left, represents the same average error between the gold standard and the reconstruction performed using the socket ball model fed by the IMU outputs only. On an average arm-length of about 80 cm, the maximum error committed in terms of distance between the real position of the hand and the estimation, as reported in Table 3, can reach an amount of about 20 cm. The heavy bias of the data corresponding to high values of the abduction angle is due to the excessive simplification of the kinematic of the scapular girdle obtained by completely neglecting the movement of the scapular-thoracic joint. The average errors committed by the two approaches with respect to the gold standard are compared in Figure 8.

**Table 2:** **Mean errors between the reference trajectory (relieved by the gold standard system) and the hand posture reconstruction generated by *F* on vertical middle planes according to different horizontal flexions and abductions (five people, three trials)**.

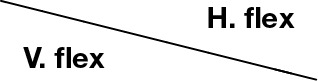	10°	20°	30°	40°	50°	60°	70°	80°
20°	0.7	0.9	1.3	1.6	1.0	1.3	1.1	0.9
40°	1.8	1.9	1.6	2.2	1.5	1.5	1.4	1.2
60°	1.6	1.6	2.5	2.4	2.2	1.6	1.3	1.8
80°	1.3	2.2	2.4	2.9	2.6	2.1	1.9	2.1
100°	2.1	2.8	2.8	3.1	3.3	2.4	2.1	1.9
120°	2.6	3.0	3.1	3.6	3.2	2.9	2.6	2.3

**Table 3:** **Mean errors between the reference trajectory (detected by gold standard system) and the hand posture reconstruction generated by the socket ball and IMU reconstruction on vertical middle planes according to different horizontal flexions and abductions (five people, three trials)**.

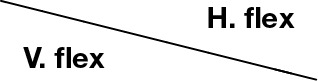	10°	20°	30°	40°	50°	60°	70°	80°
20°	0.8	1.2	1.4	2.2	1.8	1.6	1.2	0.7
40°	1.6	2.2	2.5	2.3	2.4	2.1	1.5	1.2
60°	2.6	2.6	3.0	3.2	3.1	2.7	2.4	2.1
80°	4.5	4.9	5.8	6.1	5.6	5.5	4.7	4.3
100°	8.5	9.7	9.8	10.9	11.2	10.5	9.3	7.5
120°	19.4	20.6	21.3	22.44	22.7	22.0	19.3	18.2

**Figure 9:**
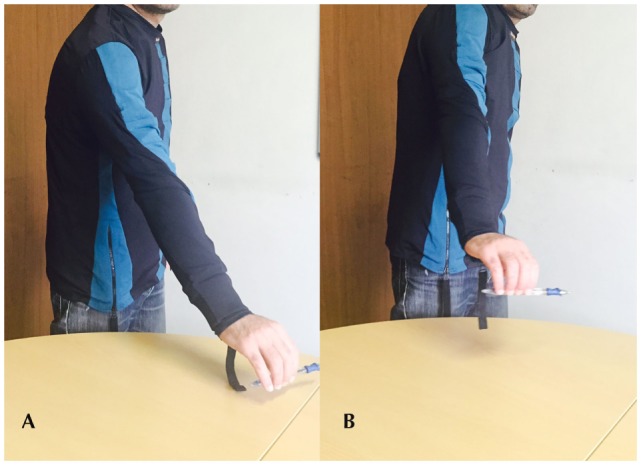
**The execution of a reaching movement (A) in physiological condition, by the correct use of the deltoid muscle (B) performed by a compensatory movement thanks to the trapezius muscle**.

Depending on the degree of stroke impairment, the control of the upper and lower limb muscles can either be lost, limited by fatigue due to the execution of a few tasks, or the functional capacity remains unchanged. Generally, the distal part is most at risk of losing motor control. When muscle deafferentation (i.e., the incapacity of a nerve to recruit muscle fiber) occurs, a typical body attitude is evident due to the dominance of flexor muscles (which are in major numbers and often more powerful) together with inadequate contractions of antigravity muscles. This phenomenon may be reversible by patient training, and the use of a correct motor scheme can often be recovered. In several cases, the degree of recovery is estimated according to the progress in the ability to perform certain movements. One of the most evident impairments produced by stroke on the upper limb can be the deafferentation of the deltoid muscle (or its early fatigue after a few tasks). In reaching movements, for example, the arm abduction and flexion of the gleno-humeral joint are often replaced by a scapular elevation followed by a scapular abduction (Figure 9). This happens because while the first movement involves a strong action of the deltoid, the second one uses the trapezium, serratus anterioris, and pectoralis major that are more proximal muscles from an innervation point of view and, thus, are more controllable. The task is completed, in most cases, but in the second case the pathological synergy induces a set of secondary illnesses (inflammation, usury illness, or impingement).

**Figure 10:**
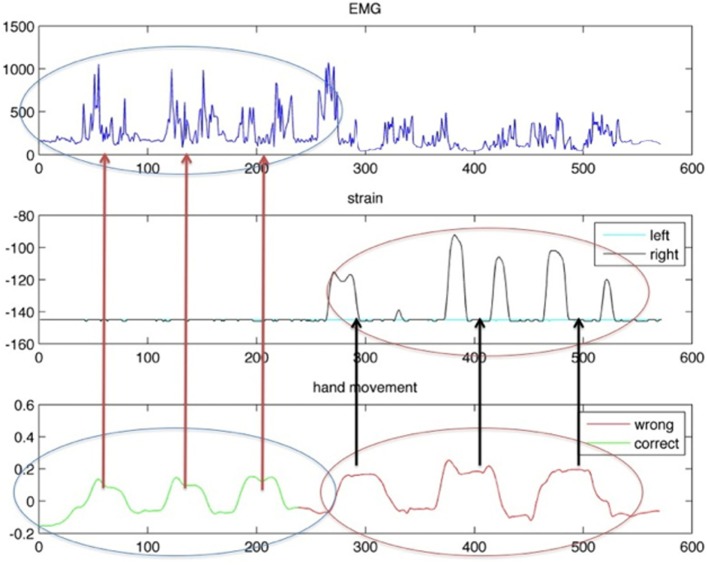
**Behavior of IMU on the arm (on the bottom), strain sensor (in the middle), and EMG response (on the top)**. On the left, data acquired during a correct reaching movement. On the right, data derived from a compensatory movement are plotted.

If we detect the two movements using only the IMU information, in practice we obtain an identical reconstruction. It is clear that the two movements are indistinguishable if the scapula movement is not taken into account and progress in recovery remains undetected if we only use this information. By inserting data derived from both the EMG electrode and the strain sensor, it is possible to distinguish the two movements. As presented in Figure 10, on the left, when the correct abduction of the arm is executed, corresponding to an IMU output (green, on the bottom), the strain sensor (in the middle) placed on the scapula provides no output. Simultaneously, the EMG electrodes (at the top of the graph) placed on the deltoid muscle detect any activity correlated to the muscle activation that moves the gleno-humeral joint. On the other hand, when the compensatory movement of scapula elevation and rotation is executed, the IMU system relieves the same behavior (in red, on the bottom right) but the strain sensor detects a movement of the scapula with respect to the sternum and rib cage (in the middle right of the figure).

### 4.2 Module 2: Sensing Trousers

The sensing trousers were conceived for the ambulatory evaluation of kinematic asymmetries between affected and unaffected legs in walking activity. According to the INTERACTION sensing architecture reported in Figure 1, the gait kinematic can be detected using the IMUs on the thighs and shanks combined with the sacrum and shoe IMUs. An additional requirement of the INTERACTION sensing system was the estimation of the body center of mass (CoM) movement relative to the center of pressure during daily-life gait. To obtain this parameter, the sensing trousers were designed to detect the relative foot position to be used in combination with the shoe pressure measurements following the approach described by Schepers et al. (2009).

#### 4.2.1 Sensing Trousers: Methods

As shown in Figure 1, KPF goniometers were included in the knee areas to improve the quality of the movement detected through data fusion with the IMUs and to potentially reduce the system complexity and reduce the overall cost of the prototype. Indeed, given that the prototype will be used by post-stroke survivors, it would be very advantageous to reduce the overall system complexity and increase the usability. Our objective in this study was to demonstrate that thigh and shank IMUs – in terms of knee flexion-extension estimation – do not add information with respect to the textile knee sensor. This will enable designers to remove the more expensive sensors, thus obtaining a cheaper and more comfortable device. The electronic devices specifically designed for the acquisition of the KPF knee goniometers were connected to the thigh IMU board (XSens MTw) and integrated into a dedicated casing (Figure 1 on the left). To compare the KPF goniometer output and the knee flexion-extension estimation performed by the thigh and calf IMUs, data were acquired on different motor tasks, such as knee flexion-extension in monopodalic contralateral standing position and slow, medium, and fast speed walking. For each motor task, the experiments were repeated five times. The KPF goniometers were calibrated according to the procedure described in section 3.2 in order to measure 0° when the knee was completely extended. Regarding the IMU system, the knee flexion-extension angle was extracted taking into account the components of the rotation matrix that describe the orientation of the IMU frame on the calf with respect to the frame of the IMU placed on the thigh.

#### 4.2.2 Sensing Trousers: Results

**Figure 11:**
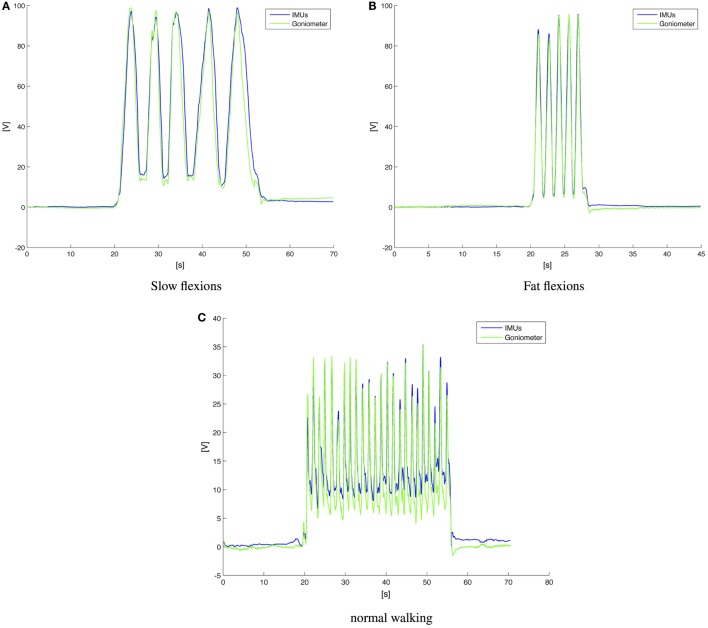
**Comparison between goniometers and IMUs in knee flexion extension detection**. **(A)** Slow knee flexion during monopodalic standing; **(B)** fast knee flexion during monopodalic standing; and normal speed walking velocity **(C)**.

The knee flexion-extension measured with the KPF goniometer *θ_g_*(*t*) was compared with the angle obtained by the thigh and shank IMUs *θ_IMU_*(*t*). A preliminary graphical evaluation of the performance comparison is reported in Figure 11 for three representative plots of slow knee flexion (Figure 11A), fast knee flexion (Figure 11B), and walking at normal velocity (Figure 11C). Both in the slow/fast knee flexion-extension and in walking activity, the double layer KPF goniometer shows a good performance in angular measurements and was able to follow dynamic knee movements. To reinforce this initial analysis, a statistical approach was followed. We used a statistical inferential *t*-test to determine whether the samples obtained by the two different measurement systems *θ_g_*(*t*) and *θ_IMU_*(*t*) belonged to the same population. The two statistics of equations (12) and (13) were considered for this analysis.

(12) X=θIMU−θg¯=1t1−t0∫t0t1 θIMU(t)−θg(t)dt,

(13) σ=||θIMU−θg||2=1t1−t0∫t0t1 θIMU(t)−θg(t)2dt.

Assuming that *θ_IMU_*(*t*) and *θ_g_*(*t*) are two random variables, the difference *X* = *θ_g_*(*t*) − *θ_IMU_*(*t*) also has to be a random variable. If we prove that *X* is a zero-mean random variable that respects the chosen confidence *σ*, we have proved that the system is redundant and one sensor can be removed. In practice, the described test (performed as a parametric Student test) identifies whether or not the data detected by the goniometer belong to the trial population using the couple of IMUs. With a significance level of *σ* = 0.05, the *t*-tests, performed on the zero mean variable *X* associated with the SD *σ*, produced the results shown in Table 4. In each of the examined cases, the zero-hypothesis is verified, i.e., the goniometer and the couple of IMUs provide the same information with the chosen confidence. Considering the INTERACTION prototype, this implies that, from a minimal-sensor-system point of view, the IMU on the calf does not provide any additional information on the knee flexion. The calf IMU could possibly be removed, since the IMU placed on the shoe provides information on the knee torsion (no movements on the horizontal plane are allowed by the ankle and the 3D info on the feet shows the intra-extra rotation of the femoral-tibial joint).

**Table 4:** **Statistics on differences between IMU and goniometer behavior**.

Activity	*X*	*σ*	*t*	*p*	Verified
Slow flexion	0.06	0.28	−1.56	0.33	Y
Normal flexion	−0.04	0.33	0.93	0.36	Y
Fast flexion	0.08	0.40	−1.63	0.20	Y
Slow walking	0.07	0.30	−1.61	0.26	Y
Normal walking	−0.06	0.39	0.89	0.28	Y

*t represents the student statistic, and *p* is the related *p*-value*.

### 4.3 Module 3: Sensing Glove

**Figure 12:**
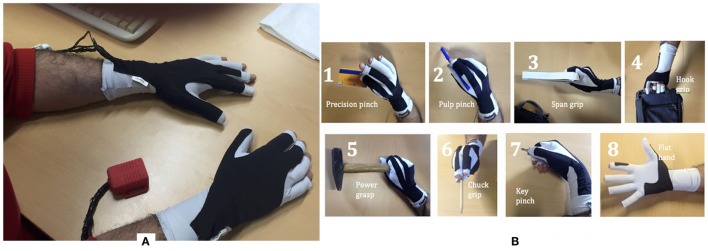
**(A)** Kinesthetic and kinetic glove pair; **(B)** functional hand grips to be classified.

The sensing glove was developed for the ambulatory evaluation of the residual hand function and its recovery, as briefly outlined in the Introduction. We developed both left and right hand gloves to simultaneously monitor the affected and un-affected arms (Figure 12A). The sensing glove is characterized by kinesthetic and kinetic parts with different functionalities.

#### 4.3.1 Sensing Glove: Methods

**Figure 13:**
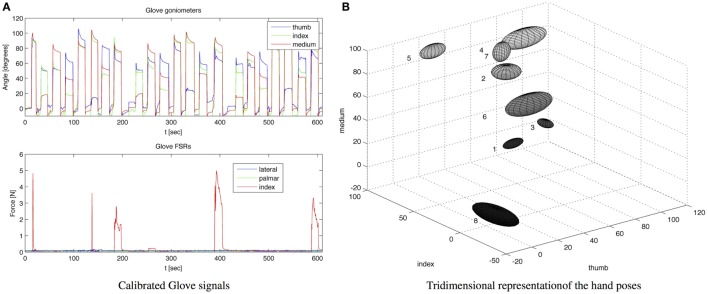
**(A)** Raw data measured by the calibrated glove: goniometer angles are reported in the above picture, FSR force measurements are reported in the picture below; **(B)** Tridimensional representations of the acquired hand poses in the space of the goniometer outputs. For each hand pose, an ellipsoid is plotted – with the center in the mean pose and semi-axes twice the pose SD.

The kinesthetic part of the gloves, which assess the patient grasping activity, consists of three KPF goniometers directly integrated into the elastic lycra^®^ fabric. For index and middle fingers, in order to detect the flexion-extension movement the KPF goniometers were placed on the dorsal side of the hand in relation to each metacarpal-phalangeal joint. To capture the opposition of the thumb, the goniometer was placed in relation to the trapezium-metacarpal and the metacarpal-phalangeal joints. In one of our previous works, the glove goniometers were compared with a gold standard instrument (Smart DX 100 produced by BTS Bioengineering^2^) and showed angular errors in the order of a few degrees (Carbonaro et al., 2014). To improve the performance in grasping an additional FSR sensor was integrated into the lateral side of the index finger (i.e., pose 2 *pulp pinch* and seven *key pinch* resulted to be very close in the *R*^3^ space represented by the three goniometer outputs). This minimal sensor configuration – with a low number of monitored degrees of freedom – is a trade-off between a good grasping discrimination and design constraints typical of wearable and ambulatory applications. Indeed, the number of KPF goniometers needs to be low to increase the wearability and user comfort through a reduction in the number of sensors and interconnections (i.e., an individual goniometer has six connecting wires as highlighted by the authors in Carbonaro et al. (2014)). In addition, the high number of wireless modules in the INTERACTION sensor network (14) introduced additional design constraints in favor of a reduction in the sensor number (limited bandwidth available for the streamed glove data and low power consumption). Despite the low number of goniometers, the information deriving from thumb, index, and medium finger is sufficient and the basic hand grips (Lister, 1977) are differentiated. In addition, studies on hand synergies have demonstrated that monitoring a low number of relevant joints leads to an efficient grasping discrimination (Bianchi et al., 2013, 2014). The kinetic part of the glove, which was designed to evaluate patient force interaction with the environment, is made from five FSR sensors integrated in the glove palm (two sensors) and lateral side (three sensors) in order to measure contact pressures and provide an adequate indication of hand loading. The FSR sensor locations are represented by the yellow pads sketched on the left of Figure 1 in the glove module. A dedicated processing algorithm analyses the FSR sensor outputs to extract a three-state indicator of force interaction (no contact, mild force, high force). The electronic device specifically designed for the acquisition of the kinesthetic and kinetic glove data was connected to the IMU board on the hand (XSens MTw) and integrated into a dedicated casing (red box of Figure 13). The IMU/Glove board, placed on the dorsal side of the hand and represented in Figure 1 by the orange box on the glove module, detects the overall orientation and movement of the hand, which is useful for additional observations of reaching activities in combination with the other shirt sensor readings.

#### 4.3.2 Sensing Glove: Results

**Figure 14:**
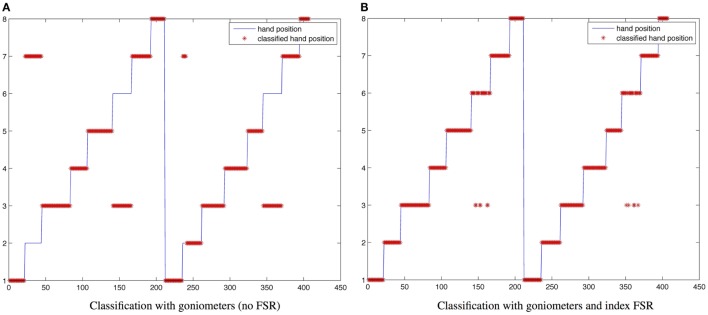
**Classification results**. **(A)** With goniometers without the index FSR; **(B)** with goniometers and the index FSR.

The sensing gloves were used to develop the hand pose classifier for the recognition of the eight fundamental hand positions described by Lister and reported in Figure 12B. In order to evaluate the ability of the glove to recognize the hand poses considered, hand positions were acquired several times from five subjects. The glove goniometers were calibrated according to the procedure described in section 3.2 by reading the sensor outputs in the flat hand position and with the hand fully closed and associating 0° and 90° with these two poses. Figure 13A shows the calibrated goniometer data acquired while the subject performed three repetitions of the eight poses in Figure 12B, starting from position 1 and ending at position 8. The data acquired were also represented in the three-dimensional space of the goniometer outputs in order to show the data spread among the different hand poses, as shown in Figure 13. For each position, this figure represents an ellipsoid with the center in the mean angles of the pose and the lengths of the semi-axes twice the SD such as if the angular measurements include the *i*-th ellipsoid, the pose is likely to be the *i*-th one. As shown in Figure 13, the poses are well separated. In addition, the figure highlights the action of the index force sensor that detects a significant force while the subject manipulates the object by the lateral side of the index finger (e.g., *key pinch* – position 7 of Figure 13B). Considering the results shown in Figure 13, an *ad hoc* algorithm aimed at classifying hand poses was developed and tested. The *i*-th position is recognized if the goniometer measurement is contained in an ellipsoid with the center in the mean pose and the semi-axes three times the SDs. If the angular measurements are outside each of the eight ellipsoids, no position is recognized. Results of the classification algorithm are reported in Figure 14A for a sample acquisition.

As expected, due to the reduced sensor set, the results are reasonably accurate, but there are errors in discriminating between position 2 (*pulp pinch*) and position 7 (*key pinch*), since these positions are very close in the *R*^3^ space spanned by the three sensor outputs. The same classification approach was applied, including the measurement of the FSR sensor placed on the index finger, in this case we considered ellipsoids in a *R*^4^ space. As observed from Figure 14B, there is no misclassification between positions 2 and 7. Some errors still exist in discriminating between positions 3 and 6 (i.e., also these positions are very close), but the overall performance is very good.

## 5 Discussion

Tests performed in the final phase of the INTERACTION project provide important new insights into measuring motor performance in stroke subjects during daily activities. The present work, which describes the sensing platform and a technical evaluation of the prototype, focused on three particular aspects of the innovation introduced within the project. The first aspect concerns the improvement in the estimation of the hand position during reaching activities. The results show a notable improvement in the determination of the hand position (and consequently its trajectory) with respect to previous widely used systems and based on the modeling of the shoulder as a simple socket ball joint. The introduction of a bi-articular model for the shoulder, based on classical kinesiological principles, and taking into account the scapulothoracic joint as a separate articulation, led to an innovative approach to solve the best estimation issue, using wearable sensors alone. The results were compared with an optical system, highlighting a considerable error reduction derived from the sensor fusion among IMUs and the strain sensor with respect to the estimation performed by inertial sensing alone. In addition, the introduction of the EMG electrode on the deltoid led to the discrimination of a physiological and compensatory motor scheme in shoulder movements during hand reaching. The second module consists in a pair of trousers. The specifications of the project required a complete monitoring of the lower limb. The system was designed, including two IMUs applied on the femur and the shank. In addition, a textile goniometer was placed on the knee, making the system redundant. This work proved that, the information derived from the entire set of sensors and a reduced platform comprising only a IMU (on the thigh bone) and the goniometer is the same. From a practical point of view, this reduces the encumbrance of the instrumentation placed on the trousers, and decreases the manufacturing cost thanks to the cheapness of textile sensors. Finally, the third module presented is the sensing glove, which respected the project specifications. This glove needed to be capable of identifying the type of grasp, and the device discriminated between all the selected poses using only three KPF goniometers placed on three fingers and an additional force sensor, which improved the identification of the precision pinch and key pinch. Two force sensors placed on the palm and the medial side of the hand detected the type of interaction of the hand with the environment. This is derived from the need in stroke rehabilitation to understand if the hand is used to help leg activities (e.g., as a support in standing up). The glove was also capable of reconstructing angles for metacarpal and proximal carpal joints, although this task was not included in the project specifications.

## 6 Conclusion

This paper has described the development and laboratory assessment of a wearable textile-based sensing platform, specifically designed for the daily life evaluation of stroke patient recovery and residual functionality. The platform – developed within the EU project INTERACTION – was conceived as an unobtrusive sensing system capable of fulfilling both clinical requirements and end-user needs (i.e., easy to put on/take off, usability, comfort). These requirements were formalized as objective criteria or *metrics*: reaching distance, sternum orientation, smoothness of the hand movement, hand grasps performed, temporal and kinematic gait parameters, weight distribution, and balance. To maximize user comfort and make the system really usable in daily life, fabric-based deformation and flexion sensors (KPF technology), textile EMG electrodes, and flexible pressure sensors (FSR) completed the biomechanical information derived from a set of IMUs. We introduced three main sensing modules for evaluating stroke patient functionality of the main body regions: a shirt (i.e., upper limb, reaching activity), a glove (i.e., hand, grasping), and trousers (i.e., lower limbs, gait). For each module, we described the sensor architecture and the related reconstruction and fusion (i.e., between textile and non-textile sensors) algorithms. We tested the described sensing modules in laboratory sessions on healthy subjects to provide a first but important technical pre-clinical validation of the wearable prototype. Our tests showed the good performance of the developed prototype that was able to fulfill the initial requirements. Clinical tests on stroke patients were beyond the scope of our research and were performed in a subsequent phase using the same wearable prototype described and tested in our work (van Meulen et al., 2016). This clinical experience highlighted that different reduced subsets of the criteria defined for the upper and lower extremities may be important for the daily-life motor performance assessment of individual stroke subjects, depending on the individual’s condition. As a future work, we will exploit a number of reduced sensor systems that can be easily applied with minimal obtrusiveness for daily-life assessment of subsets of recovery-evaluation functionalities, according to the defined metrics. In particular, the use of a reduced set of sensors that maintain the same information, as highlighted for the knee sensor system within this work, will be investigated both from a cost-saving and lack of obtrusiveness perspective.

## Ethics Statement

The study protocol is a subset of a larger protocol developed within the INTERACTION (FP7-ICT-2011-7-287351) project and was carried out in accordance with the recommendations of Swiss medical and ethical committee. All participants were healthy subjects and gave written informed consent in accordance with the declaration of Helsinki.

## Author Contributions

FL and AT drafted the manuscript. FL, AT, and NC performed the experimental trials. NC, RP, PV, and DR revised the paper.

## Conflict of Interest Statement

The authors declare that the research was conducted in the absence of any commercial or financial relationships that could be construed as a potential conflict of interest.

## Footnotes

^1^http://www.xsens.com/en/general/mtw

^2^Bts. Available at: http://www.btsbioengineering.com/products/kinematics/bts-smart-dx/
